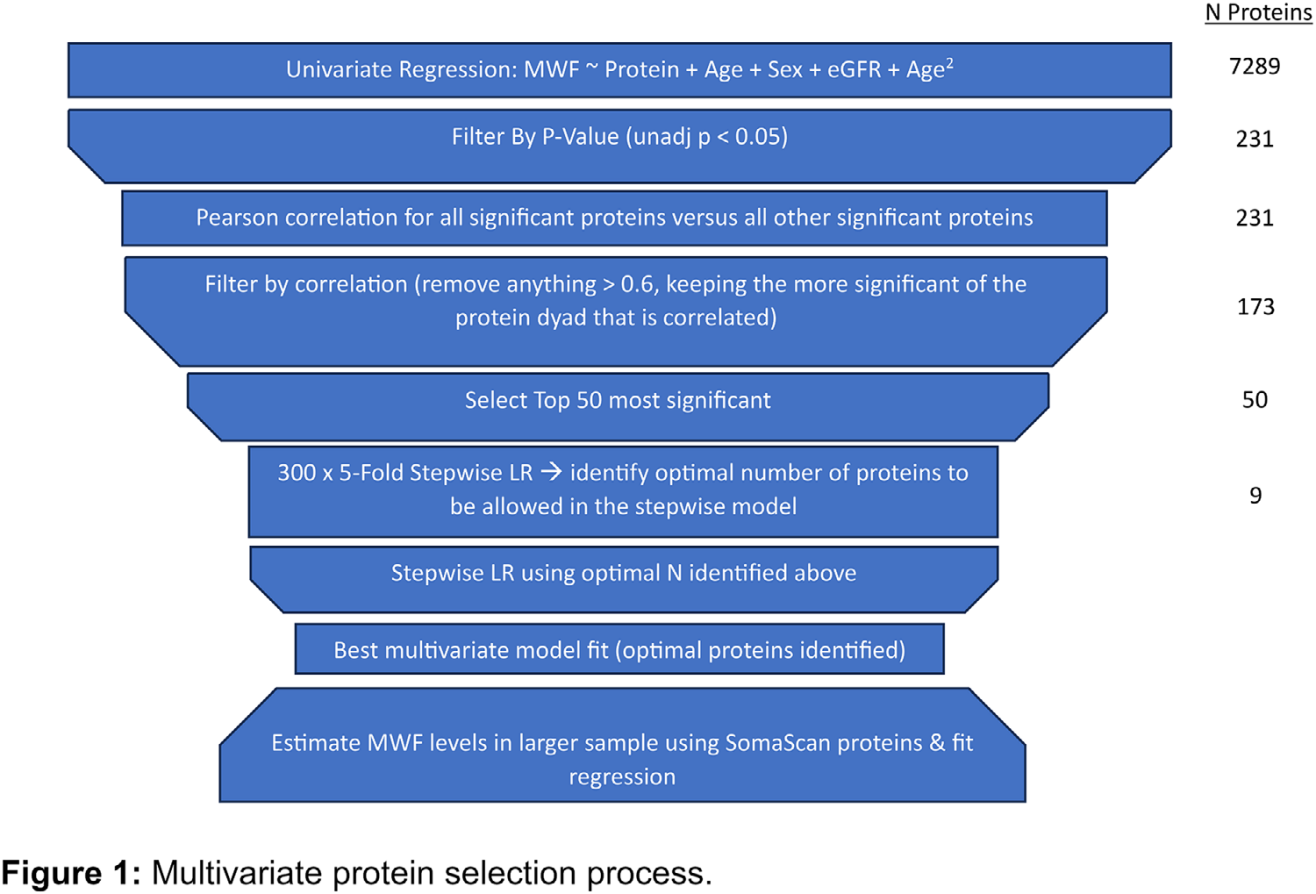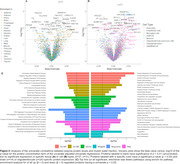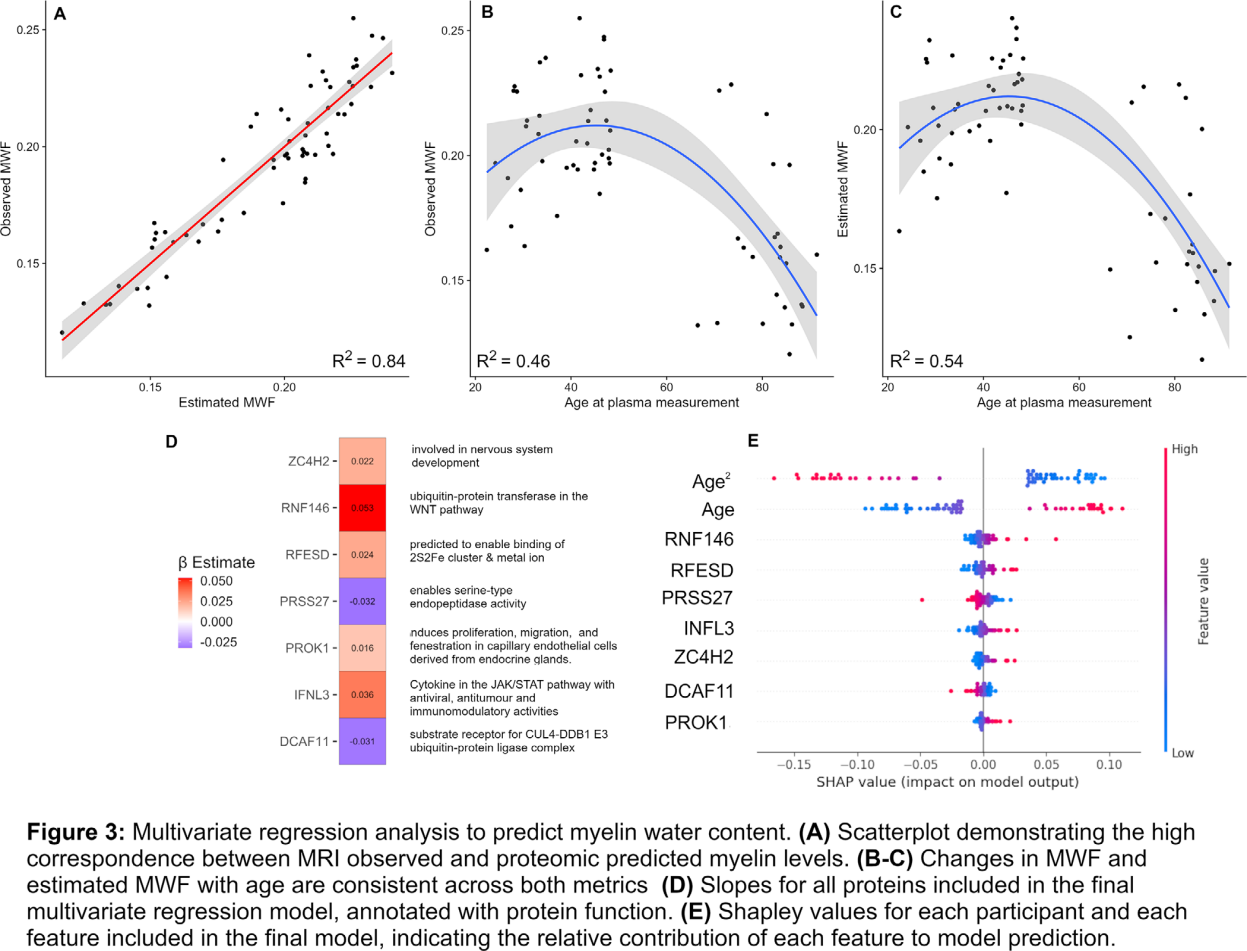# Proteomic prediction of myelin water fraction using the SomaScan 7k platform

**DOI:** 10.1002/alz.095556

**Published:** 2025-01-09

**Authors:** Cassandra M Joynes, Susan M. Resnick, Mustapha Bouhrara, Keenan A. Walker

**Affiliations:** ^1^ Laboratory of Behavioral Neuroscience, National Institute on Aging, Intramural Research Program, Baltimore, MD USA; ^2^ Laboratory of Clinical Investigation, National Institute on Aging, Intramural Research Program, Baltimore, MD USA

## Abstract

**Background:**

Myelin loss occurs as part of neurodegenerative and neuroinflammatory conditions. Myelin water fraction (MWF), a surrogate of myelin content, can be estimated using multicomponent relaxometry MRI. In this study we investigate the relationship between 7289 plasma proteins and MWF to derive a protein‐based indicator of myelin content.

**Method:**

In the Baltimore Longitudinal Study of Aging (BLSA) plasma proteins were measured using the SomaScan proteomic platform; BMC‐mcDESPOT was used to measure MWF. Univariate analyses adjusted for age, sex, eGFR, and age^2^ examined the association of protein levels with MWF. Public datasets were used for determination of the cell (HPA)‐ and tissue (GTex)‐specificity used for candidate protein annotation. Multivariate analyses used stepwise linear regression, following the selection process described in **Figure 1**, to generate a protein‐based estimate of MWF (eMWF).

**Result:**

64 cognitively normal participants from the BLSA (mean age at MWF visit 55.32 years [range 22.4 to 94.4], 48% males) were included in the discovery analysis. Adjusted univariate analyses found 231 proteins associated with MWF at an uncorrected p<0.05. However, none survived FDR correction. Plasma proteins CUTC (β = 0.02, p = 0.0003), COL20A1 (β = 0.04, p = 0.001), and PRSS27 (β = ‐0.03, p = 0.001) showed the strongest association with MWF (**Figure 2A**). Proteins positively associated with MWF were enriched for vasoconstriction, MAPK integrin signaling, amyloid‐beta binding, and complement and coagulation cascades. Proteins inversely associated with MWF were also enriched for complement and coagulation, as well as TRAIL signaling, and TP53 transcriptional regulation (**Figure 2B**). In a multivariable analysis of MWF, stepwise linear regression selected the proteins PRSS27, ZC4H2, IFNL3, RNF146, RFESD, PROK1, and DCAF11, as well as age and age^2^, for model inclusion (protein function annotated in **Figure 3D**). R^2^ of the model when fit on the full dataset was 0.86 (fivefold cross validation R^2^ = 0.51, **Figure 3A**). Observed and estimated MWF both show similar nonlinear trajectories with age (**Figure 3B‐C**).

**Conclusion:**

We developed a protein‐based proxy for total myelin content and validated the predictive ability of this measure. Future studies will test eMWF in neurodegenerative and demyelinating conditions.